# Biomarkers in immunonutrition programme, is there still a need for new ones? A brief review

**DOI:** 10.3332/ecancer.2015.546

**Published:** 2015-06-18

**Authors:** Patrice Forget, Garazi Echeverria, Simone Giglioli, Brigitte Bertrand, Stephane Nikis, Jean-Paul Lechat, Marc De Kock

**Affiliations:** 1 Departments of Anesthesiology, Cliniques Universitaires Saint-Luc, Institute of Neuroscience av. Hippocrate 10-1821, Brussels 1200, Belgium; 2 Department of Dietetics, Cliniques Universitaires Saint-Luc, Université Catholique de Louvain, Brussels 1200, Belgium; 3 Grand Hopital de Charleroi, Grand’Rue 3, Charleroi 6000, Belgium; * Equal contributors

**Keywords:** immunonutrition, inflammation, biomarkers

## Abstract

**Background and aims:**

Pre-existing malnutrition is one the most important factors affecting postsurgical complications, especially in cancer patients. The consequences of this on the immune function as well as on outcome could be reversed by immunonutrition. To help the clinician as a researcher, a routinely available biomarker (derived from clinical or biological data) would be of great importance.

**Methods:**

We reviewed the potential markers that may routinely be used in perioperative immunonutrition programmes. A comprehensive approach was used to identify and discuss the potential markers, focusing on body mass and serum biomarkers.

**Results:**

Body mass (including weight loss and body mass index) are predictive of complications, but not specifically to malnutrition. Serum markers, such as albumin, transthyretin, white blood cells counts, and C-reactive protein are not more specific. Composite scores, including the Nutritional Risk Index (NRI), the Prognostic Inflammatory and Nutritional Index (PINI), the modified Glasgow Prognostic Score (mGPS), the neutrophil-to-lymphocyte ratio (NLR), CD4 and CD8 lymphocytes counts, the platelet-to-lymphocyte ratio (PLR), the Prognostic Index (PI), and the Prognostic Nutritional Index (PNI) are prognostic factors of outcome, but are not always correlated to immunonutrition effect.

**Conclusions:**

In conclusion, there remains a lack of efficient and widely available monitoring of the effects of immunonutrition. To predict and monitor the effect of immunonutrition on immunity, efforts should be directed to the validation of routinely available tools to aid the implementation of advanced immune monitoring (like lymphocytes subpopulations counts) in clinical practices.

## Introduction

First described in 1939, in patients undergoing major surgery (especially cancer patients and also all those undergoing intraperitoneal, intrathoracic prosthetic surgeries, which are increasingly proposed to older patients), pre-existing malnutrition is one of the most important factors affecting outcome [1]. Malnutrition decreases both cell-mediated and humoral immune response, increases the risk of infections, and affects wound healing, but can be reversed by re-nutrition [1, 2, 3, 4]. This is the basis of the concept of immunonutrition, with nutrients influencing immune cell functions and inflammatory pathways [5, 6]. These immunoactive nutrients are ω-3 polyunsaturated long-chain fatty acids, arginine, and antioxidants such as ascorbic acid [5]. Turnock *et al* showed that this type of immunonutrition in patients undergoing head and neck cancer surgery may decrease circulating pro-inflammatory cytokine concentrations, and increase postoperative anti-inflammatory mediators such as IL-10 [7].

A growing body of evidence supports the concept that an optimised nutritional support reduces the number of adverse outcomes following major cancer and non-cancer surgery [8, 9]. Importantly, the treatment effect varies depending on the patient population, the intervention, and the methodological quality of the study, including the frequent lack of an efficient biomarker able to monitor the effect of immunonutrition [10].

To improve outcome, a preoperative screening of the nutritional status and an early management of malnutrition are mandatory. Nevertheless, this screening remains difficult because there is absolutely no widely accepted criterion [11, 12].

To help the clinician as a researcher and to improve the use and the comprehension of the effects of immunonutrition, a biomarker would be of great importance. In this work, we review the potential markers that may be used to screen patients and to monitor the effect of immunonutrition in the perioperative period of major surgery.

## Potential biomarkers to detect malnutrition and to monitor the effects of immunonutrition

The nutritional assessment is most often based on body mass, including weight loss and the calculation of body mass index (BMI), the albuminemia [5, 13, 14, 15, 16, 17]. Here we show that, although many assessment tools exist, none is entirely satisfactory in terms of sensitivity and/or specificity.

### Body mass

A weight loss greater than 10% of the usual weight or during the six last months is a predictive factor of postoperative complications in major surgery [13]. Even if it a widely used marker, one can say that severe malnutrition is sometimes present in the absence of weight loss, such as with ascites because of liver disease. The BMI is another important marker for the clinical evaluation of the nutritional status. A BMI lower than 18.5 is associated with an elevation of mortality and infection morbidity in cardiac and general surgery [14, 15], but again same flaws as for weight loss can be observed. Hence, a serum marker, such as albumin could be interesting.

### Serum protein levels: albumin and transthyretin (prealbumin)

In the past, serum proteins and albumin level have been proposed as biological markers of malnutrition [5]. However, there are a lot of contradictions between the results of scientific studies and use of this marker in practice. For example, serum albumin level is not well correlated to protein mass, is affected by distribution and dilution (leading to discordance with other marker, especially after surgery), and is now more considered as a disease severity/prognostic marker as in cancer [31, 32]. Moreover, serum albumin does not always significantly increase after nutritional programmes [16]. Some authors proposed to replace it with transthyretin (prealbumin), more correlated to nutritional status [34]. Nevertheless, a role of transthyretin in the monitoring of the effect of immunonutrition was not confirmed [33].

To increase sensitivity, the combination of albumin levels with the patient body weight in the NRI was proposed [17].

### A combination: the NRI

The NRI is a composite tool that allows a quantification of the risk of malnutrition. The NRI is calculated using the formula: NRI = (1.489 × serum albumin in g/L) + (41.7 × current weight/usual weight) [18]. It has been suggested that a NRI > 100 indicates that the patient is not malnourished, while 97.5–100 indicates mild malnourishment, 83.5–97.5 indicates moderate malnourishment, and <83.5 indicates severe malnourishment [17]. The main advantage of this composite score is the potential correction of the masking effect on weight loss (i.e. absence of weight loss or even weight gain) because of oedemas in case of hypoalbuminemia. Nevertheless, this tool does not permit the monitoring of the potential therapeutic effect of nutrition, e.g. the reversing effect on immune function, whereas immunonutrition formulas have been developed to improve nutrition, cell immune function, and to modulate inflammation [10].

### White blood cells counts and C-reactive protein (CRP)

White blood cell counts (WBCs) has been tested as possible parameters for the effects of immunonutrition in patients [8, 19, 20]. Unfortunately, no significant difference in WBC between the immunonutrition group and the normal nutrition group was showed by Giger *et al* during the first postoperative week [21].

On the other side, CRP as endotoxin levels (a potent trigger of acute phase reaction) were significantly lower in the treatment group on postoperative day 7 [21, 22]. In contrast, in a cohort of 116 patients undergoing major abdominal surgery (either receiving or not receiving immunonutrition), we were not able to confirm any difference in the pre- and postoperative CRP levels and WBCs [20].

### WBC and/or CRP are not definitive solutions

The components of the systemic inflammatory response such as CRP, neutrophils, lymphocytes, and platelets, vary depending on the immune state of the patient. The Prognostic Inflammatory and Nutritional Index (PINI) combining orosomucoid, CRP, prealbumin, and albumin levels has been proposed as a solution [24, 25, 26]. Unfortunately, large and rapid changes in CRP in acute inflammation overestimate the values of PINI. Additionally, a decrease of orosomucoid is observed in hepatocellular insufficiency, acute kidney injury, with some medications (corticosteroids, NSAIDs, erythromycin, penicillin, α-and beta-blockers, sulfasalazine) and in terminal stages of some neoplasias, which makes it an unreliable parameter [25]. As a result, the question remains about the interest of other inflammatory scores in this context [27, 28]. This is an actual challenge in therapeutic and preventive cancer research to detect potential targets in chronic inflammatory disease which are essential links to promote inflammation-associated cancer [29].

### WBC-derived scores: from the neutrophil-to-lymphocyte ratio to the CD4:8 ratio

The combinations of the components of the systemic inflammatory response to form prognostic scores based on inflammation such as the modified Glasgow Prognostic Score (mGPS), the neutrophil-to-lymphocyte ratio (NLR), the platelet-to-lymphocyte ratio (PLR), the Prognostic Index (PI), and the Prognostic Nutritional Index (PNI) have been evaluated and compared in cancer research [30, 31, 32]. It is known that systemic inflammation-based scores have prognostic value in a variety of cancers. In fact, an elevated mGPS, NLR, PLR, PI, and PNI are independently predictive of a shorter cancer specific survival [31, 32]. Recently, the interest of the NLR in the context of the possible influence of immunonutrition on inflammation was assessed [23]. As neutrophils and lymphocytes counts are related with the systemic inflammatory response, independently from the liver function but linked to malnutrition and zinc deficiency, it was logical to combine neutrophils and lymphocytes counts. A score like the NLR could potentially be more sensitive than WBCs counts [32]. However, clinical data did not confirm that NLR value is a good marker of the influence of immunonutrition on inflammation [23, 31].

To overcome this problem, some authors proposed to follow lymphocytes subpopulations. In head and neck cancer, changes suggesting that postoperative preservation of specific lymphocyte functions were associated with immunonutrition [33, 34]. This was especially observed for CD3 T cells and CD4 T helper cells counts, but also considering CD8 T suppressor cells counts and CD4:8 ratio [33, 34]. Nevertheless, the use of the lymphocytes subpopulations counts remains limited in routine practice.

## How to interpret these discrepancies?

Immunonutrition is not always associated with a significant variation of the inflammatory markers, despite a presumed anti-inflammatory effect and influence on immune parameters. However, these studies have several limitations. First, looking at sensitive markers, like CRP, WBCs, or NLR in cancer patients, some of them are unavoidably outliers [20, 23]. This phenomenon can be explained, for example, by emergent surgery, high inflammatory status (e.g. in sepsis). This fact can challenge any robust analysis. Second, heterogeneity is frequent, precluding definitive conclusion before analysis on large patients’ cohorts. A typical high level of internal variability is seen in catabolic situations like after radiotherapy, chemotherapy (and tumour necrosis), immunosuppressive therapy, or changes in the distribution of leucocytes (like with the use of corticosteroids). All these limitations implicate the impracticability of the use of the parameters, described in this paper in a clinical setting. Nevertheless, we cannot definitively exclude their use in clinical research, as a condition for careful selection of patients’ population.

## Conclusion

In conclusion, there remains a lack of efficient and widely available monitoring of the effects of immunonutrition. To predict and monitor the effect of immunonutrition on immunity, efforts should be directed to the validation of routinely available tools for the implementation of advanced immune monitoring (like lymphocytes subpopulations counts) in clinical practice.

## Statement of authorship

PF, GE, SG, BB, MDK have participated to the design of the work, the selection of the sources, the analysis, the redaction, and approved the final version. SN and JPL participated in the reflexion, the critical appraisal of the sources, discussed the manuscript, and approved the final version.

## Conflicts of interest

BG received from Nestlé SA covering for travel expenses to go to one congress. The other authors attest to no conflicts of interest.

## Financial support/Funding sources

This work was exclusively supported by the Departments of Anesthesiology of the Cliniques Universitaires Saint-Luc, Brussels, Belgium.
